# Carbon monoxide regulates the expression of the wound-inducible gene *ipomoelin* through antioxidation and MAPK phosphorylation in sweet potato

**DOI:** 10.1093/jxb/eru291

**Published:** 2014-07-25

**Authors:** Jeng-Shane Lin, Hsin-Hung Lin, Yu-Chi Li, Yu-Chi King, Ruei-Jin Sung, Yun-Wei Kuo, Chih-Ching Lin, Yu-Hsing Shen, Shih-Tong Jeng

**Affiliations:** ^1^Institute of Plant Biology and Department of Life Science, National Taiwan University, Taipei 10617, Taiwan; ^2^Biodiversity Research Center, Academia Sinica, Taipei 11529, Taiwan; ^3^Institute of Plant and Microbial Biology, Academia Sinica, Taipei 11529, Taiwan

**Keywords:** Carbon monoxide, ERK phosphorylation, H_2_O_2_, ipomoelin, sweet potato, wounding.

## Abstract

Carbon monoxide (CO), one of the haem oxygenase (HO) products, plays important roles in plant development and stress adaptation. However, the function of CO involved in wounding responses is seldom studied. A wound-inducible gene, *ipomoelin* (*IPO*), of sweet potato (*Ipomoea batatas* cv. Tainung 57) was used as a target to study the regulation of CO in wounding responses. After wounding for 1h, the endogenous CO content and *IbHO* expression level were significantly reduced in leaves. *IPO* expression upon wounding was prohibited by the HO activator hemin, whereas the HO inhibitor zinc protoporphyrin IX elevated *IPO* expression. The *IPO* expression induced by wounding, H_2_O_2_, or methyl jasmonate was inhibited by CO. CO also affected the activities of ascorbate peroxidase, catalase, and peroxidase, and largely decreased H_2_O_2_ content in leaves. CO inhibited the extracellular signal-regulated kinase (ERK) phosphorylation induced by wounding. IbMAPK, the ERK of sweet potato, was identified by immunoblotting, and the interaction with its upstream activator, IbMEK1, was further confirmed by bimolecular fluorescence complementation and co-immunoprecipitation. Conclusively, wounding in leaves repressed *IbHO* expression and CO production, induced H_2_O_2_ generation and ERK phosphorylation, and then stimulated *IPO* expression.

## Summary

A reduction in CO produced by *IbHO* occurs in leaves upon wounding and causes H_2_O_2_ generation and ERK phosphorylation. Defence systems are then switched on to protect plants from herbivores.

## Introduction

Carbon monoxide (CO) is an odourless, tasteless, and colourless diatom gaseous molecule that has been widely considered to be a poisonous gas since 17th century. It can be produced by haem oxygenase (HO; E.C. 1:14:99:3) in biological systems in plants (Sjostrand, 1952; Siegel *et al.*, 1962), and acts as a physiological messenger (Shekhawat andand Verma, 2010). HO is a ubiquitous enzyme that has been identified in different organisms including bacteria, algae, fungi, animals, and plants (Terry *et al.*, 2002). HO is an evolutionarily conserved enzyme that catalyses haem degradation leading to the production of equimolar amounts of CO, biliverdin, and free iron (Muramoto *et al.*, 2002; Terry *et al.*, 2002). Recent studies indicate that HO plays multiple roles in growth, development, and the stress response. It participates in the phytochrome chromophore formation to affect photomorphogenesis and light signalling in plants (Davis *et al.*, 2001; Gisk *et al.*, 2010; Shekhawat and Verma, 2010). In addition, HO acts as a downstream mediator in auxin signalling to affect root development (Cao *et al.*, 2007; Xuan *et al*., 2007, 2008*b*; Gisk *et al.*, 2012). In soybean (*Glycine max*) and *Arabidopsis*, HO is involved in reactive oxygen species scavenging to against salinity and heavy metal-induced oxidative stresses (Balestrasse *et al*., 2008*a*, *b*; Zilli *et al.*, 2009). The addition of HO1 products, especially CO, can partially rescue the UV-C hypersensitivity in the *hy1-100* mutant (Xie *et al.*, 2012).

CO is a physiological messenger involved in various plant growth and development. It promotes root elongation (Xuan *et al.*, 2007), root hair development (Guo *et al.*, 2009), adventitious root generation (Xuan *et al.*, 2008*b*), and stomatal closure (Cao *et al.*, 2007; Song *et al.*, 2008; Xuan *et al.*, 2008*a*). In addition, CO activates antioxidant enzymes, including superoxide dismutase (SOD), catalase (CAT), and ascorbate peroxidase (APX), to protect plant from the oxidative damage induced by salt (Xie *et al.*, 2008; Bose *et al.*, 2013), cadmium (Han *et al.*, 2008; Cui *et al.*, 2012), paraquat (Sa *et al.*, 2007), and UV irradiation (Xie *et al.*, 2012). It also improves adaptation of iron deficiency in *Arabidopsis* (Kong *et al.*, 2010) and delays gibberellin-mediated programmed cell death in wheat aleurone layers (Wu *et al.*, 2011). In addition, CO induces the production of nitric oxide (NO) in plants (Xuan *et al.*, 2007; Song *et al.*, 2008; Xuan *et al.*, 2008*b*), and alters the phosphorylation of p38 mitogen-activated protein kinases (MAPK) and extracellular signal-regulated kinases (ERK) in animals (Song *et al.*, 2002; Kim *et al.*, 2005; Basuroy *et al.*, 2011; Schallner *et al.*, 2012). Thus, CO participates in not only physiological regulation but also in defence responses.

In natural environments, plants develop inducible defence systems to survive biotic and abiotic threats. During pathogenic and herbivorous attacks, plants produce a wide variety of defence-related hormones, including ethylene, methyl jasmonate (MJ), salicylic acid (SA) (Pieterse & Van Loon, 2004; Manavella *et al.*, 2008), and peptide-hormones (Ryan *et al.*, 2002), to unlock the defence-related regulatory networks. Second messengers, including NO, cytosolic calcium (Ca^2+^) (Capiati *et al.*, 2006; Chen *et al.*, 2008; Howe and Jander, 2008), and reactive oxygen species (Orozco-Cardenas *et al.*, 2001; Jih *et al.*, 2003; Le Deunff *et al.*, 2004), are generated to induce defence-related genes (El-kereamy *et al.*, 2011). Post-transcriptional regulators such as microRNAs (Lin *et al.*, 2012) and post-translational regulation including MAPK cascades (Chen *et al.*, 2008; Howe and Jander, 2008) are also involved in various defence responses.

MAPK cascades, consisting of a MAPK kinase kinase (MAPKKK), a MAPK kinase (MAPKK), and a MAPK, mediate signalling transductions in various stresses in plants. AtMPK3 and AtMPK6 can be activated after treated with flagellin flg22, a pathogen-derived peptide (Galletti *et al.*, 2011). The AtMKK1–AtMPK6 module controls the H_2_O_2_ signalling in an abscisic acid-dependent pathway to regulate stress responses (Xing *et al.*, 2008). In tobacco, wounding activates wounding-induced protein kinase, the orthologous of AtMAPK3, and SA-induced protein kinase, the orthologous of AtMAPK6, and further regulates the levels of jasmonic acid and SA (Yang *et al.*, 2001; Menke *et al.*, 2004; Seo *et al.*, 2007). The addition of an MAPKK inhibitor, PD98059, blocks the expression of *Ipomoelin* (*IPO*) induced by wounding and ethylene (Chen *et al.*, 2008).

Although the antioxidant and physiological properties of CO have been studied, the role of CO in wounding is still poorly understood. *IPO*, a wound-inducible gene from sweet potato (*Ipomoea batatas*), is induced by the application of MJ, ethylene, and mechanical wounding, and is further regulated by Ca^2+^, H_2_O_2_, and NO (Imanishi *et al.*, 1997; Chen *et al.*, 2003, 2008; Jih *et al.*, 2003). In this study, *IPO* was used as an indicator to certify the relationship between CO and the wounding response.

## Materials and methods

### Plant materials and treatments

Sweet potato (*I. batatas* cv. Tainung 57) and tobacco (*Nicotiana benthamiana*) plants were grown in a controlled environment (16h/25 °C day; 8h/22 °C night; humidity 70%; light 40 µmol photons m^−2^ s^−1^). Plants with six to eight fully expanded leaves were used in this study. Sweet potato was used for gene isolation, gene expression assay, determination of CO and H_2_O_2_, enzyme activity assay, and ERK phosphorylation analysis; tobacco was used in co-immunoprecipitation experiments. *Arabidopsis thaliana* (Col-0) was grown at 22 °C under a 16h light/8h dark photoperiod with cool fluorescent light at 100 µmol photons m^−2^ s^−1^, and 15-d-old plants were used in bimolecular fluorescence complementation (BiFC) assays.

Chemical treatments were performed based on the procedure described by Chen *et al.* (2008) and Lin *et al.* (2012). In this study, chemical reagents were purchased from Sigma-Aldrich, and tanks with 99.5% pure CO gas were purchased from Ciao Chong Gaseous Corporation in Taipei, Taiwan. All results in this study were repeated at least three times, and the similar gene expression patterns were obtained.

### CO solution preparation

CO solution preparation was performed based on the method described by Kong *et al.* (2010). CO gas was passed through 20ml water in an open tube for 30min to reach saturated solution. The saturated CO in water was treated as 100% CO solution.

### RNA isolation and quantitative real-time reverse transcription (qRT)-PCR

Total RNA was isolated from leaves that were ground in liquid nitrogen using Trizol reagent (Invitrogen) according to the manufacturer’s instructions. RNA isolated from plants was treated with DNase I (Ambion) before the reaction of Moloney murine leukemia virus reverse transcriptase (Invitrogen) with primer T_25_VN (Supplementary Table S1 at *JXB* online) at 37 °C for 90min. The cDNAs were further amplified by quantitative PCR with primer sets IPO F/IPO R, IbHO1 F/IbHO1 R, and IbActin F/IbActin R (Supplementary Table S1) to detect the expression levels of *IPO*, *IbHO1*, and *IbActin*, respectively. The amplification reactions contain 1× SYBR Green Supermix (Bio-Rad), 125nM primers, and 100ng cDNA. Data were normalized by the expression levels of the *IbActin* gene and are shown as relative expression levels. The error bars indicate standard deviation (SD) from at least three biological assays.

### Isolation of *IbHO1*, *IbMEK1*, and *IbMAPK*

The conserved domains of the *HO1*, *MEK1*, and *MAPK* genes from *Arabidopsis*, tobacco, tomato, and rice were used to search the *Ipomoea* EST and WGS databases from the NCBI to obtain putative *IbHO1*, *IbMEK1*, and *IbMAPK* genes of sweet potato. The full-length sequences of *IbHO1* and *IbMEK1* genes were then obtained using a BD SMART^TM^ RACE cDNA Amplification kit (Clontech) with primer sets IbHO1 F/IbHO1 R and IbMEK1 F/IbMEK1 R (Supplementary Table S1 at *JXB* online), respectively. IbMAPK is SPMAPK (GenBank accession no. AAD37790) and was amplified by PCR with primer sets IbMAPK F/IbMAPK R (Supplementary Table S1).

### Multiple sequences alignment and phylogenetic analyses

The protein sequences of IbHO1, IbMEK1, and IbMAPK were aligned with the related protein sequences from plants using ClustalX2. The phylogenetic trees were reconstructed using the neighbour-joining method with the MEGA5.1 program. A bootstrap test of phylogeny was performed with 1000 replicates.

### CO determination

Detection of CO content was performed based on the method described by Chalmers (Chalmers, 1991; Kong *et al.*, 2010) with minor modifications. Leaves (0.5g) were ground in liquid nitrogen, added to 5ml of H_2_O, and sealed in tubes. After centrifuged at 3000*g* for 5min, supernatants (0.5ml) of samples were mixed with 0.5ml of 1mg ml^–1^ haemoglobin (Hb), which was dissolved in 0.24M ammonia solution. After 0.1ml of 0.2g ml^–1^ freshly prepared sodium dithionite solution was added, the solution was stood for 10min and then analysed. The absorbance at 595nm of the solution was measured to calculate protein concentration, and absorbance at 420 and 432nm was measured for CO content determination as described previously (Chalmers, 1991; Kong *et al.*, 2010). The CO content in the wounded leaves was represented as the values relative to those of the unwounded leaves.

### H_2_O_2_ determination

H_2_O_2_ content was quantified by the titanium chloride method as described previously (Jana and Choudhuri, 1982).

### APX, CAT, and peroxidase (POX) activity assays

APX, CAT, and POX activity assays were performed based on the method described by Lin *et al.* (2011) with minor modifications. Total protein was extracted with an extraction buffer [50mM sodium phosphate buffer (pH 6.8) and 1% Protease Inhibitor Cocktail (Sigma)]. In the APX activity assay, 33 μl of extracted protein was added to 967 μl of reaction mixture containing 50mM potassium phosphate buffer (pH 7.0), 0.5mM ascorbate, 0.1mM EDTA, and 1mM H_2_O_2_. In the POX activity assay, 30 μl POD extracted protein was added to 2.97ml of reaction mixture containing 50mM sodium acetate buffer (pH 5.6), 5.4mM guaiacol, and 15mM H_2_O_2_. In the CAT activity assay, 30 μl of extracted protein was added to 2.97ml of reaction mixture containing 50mM phosphate buffer (pH 7.0) and 10mM H_2_O_2_. These reaction mixtures were monitored at 290, 470, and 240nm for 1min for the APX, POD, and CAT assays, respectively. The enzyme activity was calculated as U min^−1^ μg^−1^ of protein. The enzyme activity of the H_2_O-treated leaves at the time point zero was treated as a value of 1 for determining the relative ratios of other reactions. Data are represented as mean±SD.

### Protein extraction and immunoblot analysis

Total protein was extracted with an extraction buffer [100mM HEPES (pH 7.5), 5mM EDTA, 5mM EGTA, 50mM NaF, 50mM glcerophosphate, 10mM Na_3_VO_4_, 10mM dithiothreitol, 1mM phenylmethylsulphonyl fluoride, 5 μg ml^–1^ of leupeptin, 5 μg ml^–1^ ofapotein, 1% Protease Inhibitor Cocktail, and 10% glycerol]. Protein concentration was determined using a Bio-Rad protein assay kit. Total proteins (50 μg) were separated by 12 % SDS-PAGE, and then transferred to Immobilon polyvinylidene difluoride membranes (Millipore). The membrane was probed with an anti-pERK antibody (Santa Cruz) followed by horseradish peroxidase-conjugated sheep anti-mouse IgG, and detected using an Immobilon TM Western Chemiluminescent HRP Substrate kit (Millipore).

### Protein expression and purification

The coding regions of *IbMEK1* and *IbMAPK* isolated from the cDNA library of sweet potato were constructed into pGEX6p-1 or pET21a to become pGEX6p-1-IbMEK1, pET21a-IbMEK1, and pET21a-IbMAPK by PCR with primer sets BamHI-IbMEK1 F/EcoRI-IbMEK1 R, BamHI-IbMEK1 F/HindIII-IbMEK1 R, and BamHI-IbMAPK F/HindIII-IbMAPK R (Supplementary Table S1), respectively. These constructs were transformed into the *Escherichia coli* Rosetta(DE3)pLysS strain to express IbMEK1 fused with a glutathione *S*-transferase (GST) or His tag and IbMAPK fused with a His tag. The recombinant protein GST–IbMEK1 and GST were puriﬁed using a GSTrap^TM^ FF affinity column (GE Healthcare). The recombinant proteins IbMEK1–His and IbMAPK–His were puriﬁed using a HiTrap^TM^ TALON^®^ Crude column (GE Healthcare).

### *In vitro* phosphorylation analysis

Total protein from leaves of sweet potato was extracted with extraction buffer [20mM Tris/HCl (pH 7.2) and 1% Protease Inhibitor Cocktail]. The extracted protein was incubated with reaction mixture containing 20mM Tris/HCl (pH 7.2), 10mM MgCl_2_, 0.5mM CaCl_2_, 0.5mM ATP, 2mM dithiothreitol, and 10 μg of purified IbMAPK–His. After incubation at 30 °C for 1h, ProBond™ Nickel-Chelating Resin (Invitrogen) was added to purify IbMAPK–His. After washing five times, the bound proteins were eluted and separated by 12% SDS-PAGE, and detected by immunoblotting using an anti-pERK andtibody (Santa Cruz) and an anti-His antibody (LTK Biolaboratories).

### BiFC

IbMEK1 fragments were isolated from the sweet potato cDNA library using PCR with primer sets BamHI-IbMEK1 F/SacI-IbMEK1 R and XbaI-IbMEK1 F/BamHI-IbMEK1 R (Supplementary Table S1), and inserted into pBI221 to obtain pBI221-IbMEK1 and pBI221-IbMEK1 Δstop codon, respectively. IbMAPK fragments were also isolated from the sweet potato cDNA library using PCR with primer sets BamHI-IbMAPK F/SacI-IbMAPK R and XbaI-IbMAPK F/BamHI-IbMAPK R (Supplementary Table S1), and inserted into pBI221 to obtain pBI221-IbMAPK and pBI221-IbMAPK Δstop codon, individually. The N terminus (YN) of yellow fluorescent protein (YFP) was inserted into pBI221-IbMEK1 and pBI221-IbMAPK via the *Xba*I and *Bam*HI sites to become pBI221-YN-IbMEK1 and pBI221-YN-IbMAPK, and the C terminus (YC) of YFP was inserted into pBI221-IbMEK1 Δstop codon and pBI221-IbMAPK Δstop codon by *Bam*HI and *Sac*I sites to obtain pBI221-IbMEK1-YC and pBI221-IbMAPK-YC, respectively.

*Arabidopsis* protoplast isolation and transformation were performed according to Yoo *et al.* (2007), and incubated at room temperature for 16h. Confocal laser-scanning microscopy was used to visualize the fluorescent signal from the protoplasts. The protoplasts co-transformed with plasmids encoding YN and YC were used as negative controls.

### GST pull-down assays

GST pull-down assays were performed according to Yu *et al.* (2011). The recombinant protein GST–IbMEK1 or GST was co-incubated with IbMAPK–His and GST•Bind™ Resin (Millipore). After washing five times, the bound proteins were eluted and separated by 12% SDS-PAGE, and detected by immunoblotting using an anti-His antibody (LTK Biolaboratories).

### Co-immunoprecipitation

Transient expression of GST, GST–IbMEK1, and IbMAPK–His in *N. benthamiana* leaves was performed based on the method described by Lin *et al.* (2012, 2013).The GST fragment was isolated from pGEX6p-1 (Clontech) by PCR using primer set BamHI-GST F/ SacI-GST R (Supplementary Table S1), and inserted into pBI221 to obtain pBI221-GST, whose 35S-GST- terminator fragment was then cloned into the *Hind*III and *Eco*RI sites of pCAMBIA1300. IbMEK1 and IbMAPK fragments were obtained from the sweet potato cDNA library by PCR with primer sets XbaI-IbMEK1 F/ BamHI-IbMEK1 R and BamHI-IbMAPK F/ SacI-IbMAPK R (Supplementary Table S1), respectively. IbMEK1 fragment was inserted into pBI221-GST to obtain pBI221-IbMEK1-GST, whose 35S-IbMEK1-GST-terminator fragment was then cloned into pCAMBIA1300. The IbMAPK fragment was inserted into pBI221 to get pBI221-IbMAPK. The His tag of pBI221-HC was then cloned into the *Xba*I and *Bam*HI sites of pBI221-IbMAPK to become pBI221-His-IbMAPK, whose 35S- His-IbMAPK-terminator fragment was further cloned into pCAMBIA1300 for assays.

Total proteins from infiltrated leaves were extracted with an extraction buffer [50mM Tris/HCl (pH 7.4), 150mM NaCl, 2mM MgCl_2_, 1mM dithiothreitol, 20% glycerol, and 1% Protease Cocktail Inhibitor], and incubated with GST•Bind™ Resin. After washing five times, the bound proteins were eluted and separated by 12% SDS-PAGE, and detected by immunoblotting using an anti-His antibody (LTK Biolaboratories).

## Results

### Reduction of CO content and *IbHO1* transcripts by wounding

**Fig. 1.**
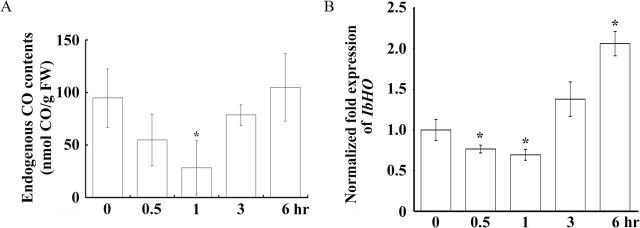
Levels of endogenous CO and haem oxygenase transcripts in sweet potato upon wounding. The third fully expanded leaves of sweet potato were wounded by forceps and collected at 0, 0.5, 1, 3, and 6h later. These leaves were used to analyse the endogenous CO contents (A) and the expression levels of the haem oxygenase gene (*IbHO*) (B). CO contents were detected by haemoglobin binding. *IbHO* expression levels were analysed by qRT-PCR. *IbActin* expression was used as an internal control. Statistic differences between unwounded and wounded sweet potato plants are marked with asterisk when *P*<0.01 according to Student’s *t*-test. The error bars are indicated as SD for at least three biological assays.

To gain insights into the regulatory roles of CO under wounding, the CO content of the unwounded and wounded leaves was analysed. CO is strongly bound by the ferrous haem in Hb). Hence, the percentage of Hb with CO was used to estimate CO content in cells. The results indicated that CO concentrations in leaves were reduced significantly at 1h and went back to normal levels at 6h after wounding (Fig. 1A). Hence, wounding may reduce CO levels in leaves temporarily.

HO is the main enzyme producing CO in plants (Terry *et al.*, 2002), and its full-length gene was obtained by rapid amplification of cDNA ends (RACE). The sequence of the IbHO1 protein showed that it is homologous to the HO1 proteins from *Arabidopsis*, *Brassica juncea*, and *Oryza sativa* (Supplementary Fig. S1A at *JXB* online), and a phylogenetic analysis of these proteins was performed (Supplementary Fig. S1B). The haem-binding pocket residues and the axial haem iron ligand of IbHO1 were also identified to the conserved residues of other HO1 in plants (Supplementary Fig. S1A). In addition, the expression of *IbHO1* transcripts upon wounding was examined by qRT-PCR in sweet potato. The expression levels of *IbHO* were decreased at 0.5 and 1h, and went back to normal levels at 3h after wounding (Fig. 1B). Taken together, the above results showed that wounding might reduce *IbHO1* expression and then decrease CO in leaves.

### Effects of IbHO1 on *IPO* expression

**Fig. 2.**
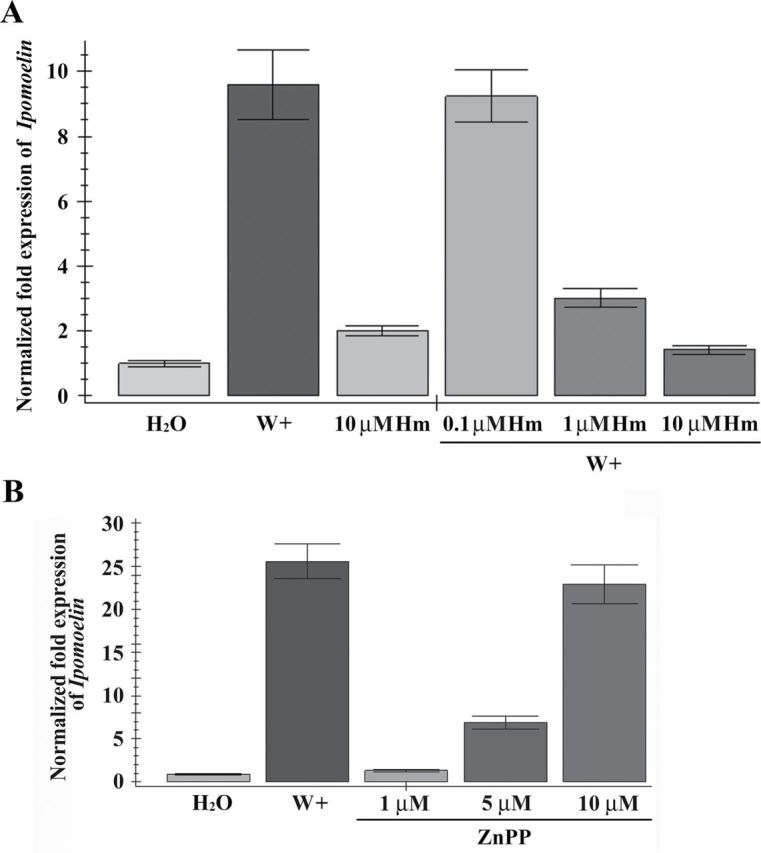
Effects of HO on *IPO* expression upon wounding. (A) Effects of the HO activator Hm on *IPO* expression. Leaves with petiole cuts of sweet potato were immersed in water for 12h and then treated with various concentrations (0, 0.1, 1, and 10 μM) of the HO activator Hm for another 12h. These leaves were unwounded or wounded (W+) by tweezers. After 6h, the total RNAs from these leaves were analysed by qRT-PCR to detect *IPO* expression. (B) Effects of the HO repressor ZnPP on *IPO* expression. The petiole cuts of leaves were immersed in water for 24h. Then, the total RNAs of leaves were isolated at 6h after wounding (W+) or the addition of various concentrations (0, 1, 5, and 10 μM) of HO repressor ZnPP. The *IPO* expression levels were analysed by qRT-PCR. The *IbActin* expression was used as an internal control. The error bars are indicated as SD for at least three biological assays.

To analyse the effects of endogenous CO on wounding responses, wound-inducible *IPO* was used as an indicator. The HO-specific activator hemin (Hm) was supplied to elevate the endogenous CO levels (Xuan *et al.*, 2007). In sweet potato, Hm can increase the CO content in leaves (Supplementary Fig. S2 at *JXB* online). Leaves of sweet potato were pre-treated without or with various concentrations of Hm for 12h, and wounded for another 6h. The *IPO* expression induced by wounding was inhibited when the solution contained 1 or 10 μM Hm (Fig. 2A). To further confirm the involvement of HO in *IPO* regulation, a potential HO inhibitor, zinc protoporphyrin IX (ZnPP), was used to inhibit the HO activity. ZnPP can decrease the endogenous CO in the leaves of sweet potato (Supplementary Fig. S2). Leaves were supplied with various concentrations of ZnPP for 6h to analyse *IPO* expression, showing that *IPO* expression was elevated in a ZnPP concentration-dependent manner (Fig. 2B). These results indicated that the activity of HO might influence *IPO* expression.

### Wounding-, MJ-, or H_2_O_2_-induced *IPO* expression is repressed by CO

**Fig. 3.**
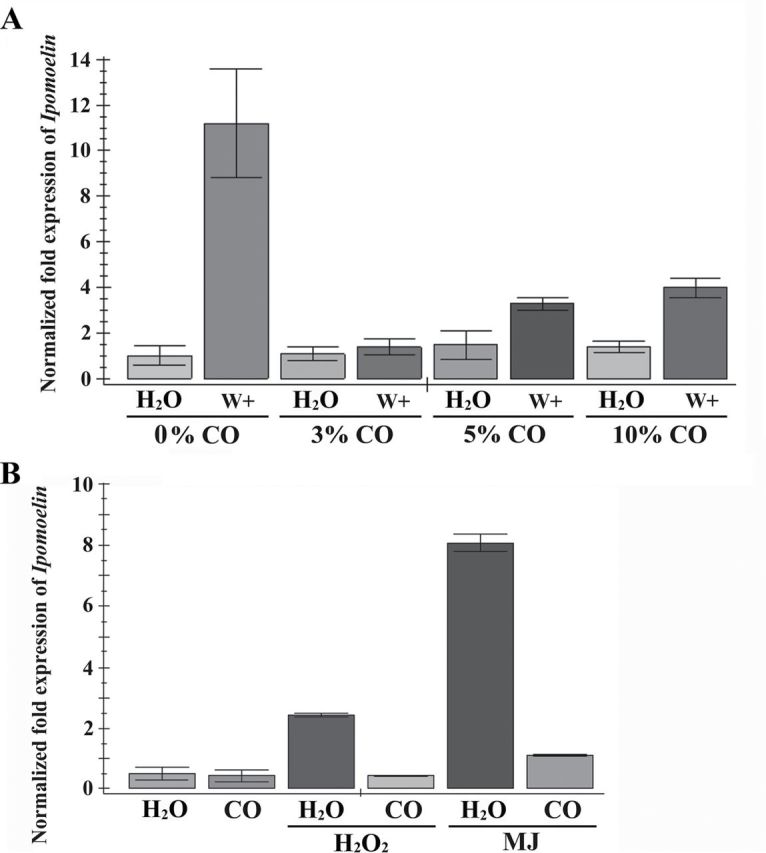
Effects of CO on *IPO* expression induced by wounding, H_2_O_2_, or MJ. (A) Effects of CO on the *IPO* expression upon wounding. Leaves with petiole cuts of sweet potato were immersed in water for 12h, and then treated with various concentrations (0, 3, 5, and 10%) of CO solution for another 12h. These leaves were then unwounded (H_2_O) or wounded (W+) by tweezers. After 6h, the total RNAs from these leaves were analysed by qRT-PCR to detect *IPO* expression. (B) Effects of CO on *IPO* expression induced by H_2_O_2_ or MJ. Leaves with petiole cuts were immersed in water for 12h and then some of these leaves were treated with 5% CO solution for another 12h. These leaves were then treated with 20mM H_2_O_2_ or 50 μM MJ for 6h. The *IPO* expression levels of these leaves were analysed by qRT-PCR. The *IbActin* expression level was used as an internal control. The error bars are indicated as SD for at least three biological assays.

Saturated CO water, prepared from bubbling CO gas through water for 30min, was used to supply CO in this study. When the leaves were pre-treated with CO water, the wound-induced *IPO* expression was inhibited in the presence of 3, 5, or 10% CO (Fig. 3A). Based on the CO level in Fig. 1A, the CO content in the leaves of sweet potato upon wounding for 1h was 29 µM when leaves contained 92% water. Because the solubility of CO in water at 25 °C is about 0.025g l^–1^ (http://www.engineeringtoolbox.com/gases-solubility-water-d_1148.html), 5% CO water was 46 µM CO. The effect of CO on sweet potato was then studied in the presence of 5% CO combined with the endogenous CO in the following studies.

In the signalling pathway of *IPO* induction, MJ and H_2_O_2_ also act as important transducers (Chen *et al.*, 2003). To monitor the function of CO in the *IPO* induction pathway, *IPO* expression in the response of MJ or H_2_O_2_ was examined in the presence of CO. The leaves were pre-treated with 5% CO for 12h, and then MJ or H_2_O_2_ was supplied for another 6h before analyses (Fig. 3B). The expression levels of *IPO* were induced by MJ or H_2_O_2_ but could not be elevated in the presence of CO (Fig. 3B). These results indicated that the presence of CO prohibited the *IPO* expression induced by not only wounding but also MJ or H_2_O_2_.

### CO reduces wound-induced H_2_O_2_ through antioxidants

**Fig. 4.**
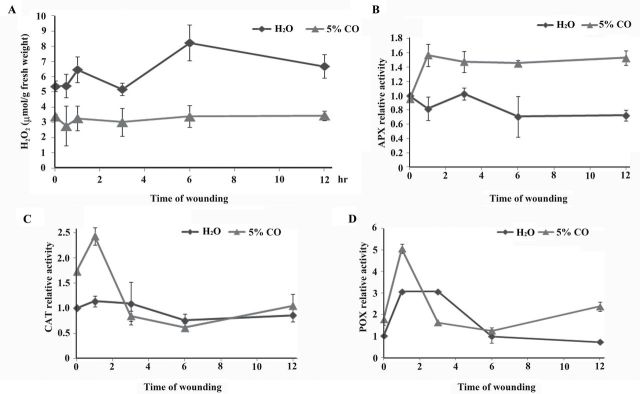
Effects of CO on the production of H_2_O_2_ and the activities of APX, CAT, and POX. Leaves with petiole cuts of sweet potato were immersed in water for 12h and then treated with water or 5% CO solution for another 12h. These leaves were then wounded by tweezers. The leaves at the times indicated were collected to extract H_2_O_2_ and total proteins. H_2_O_2_ content was detected by TiCl_4_ method (A). Total proteins were used for the activity assays of APX (B), CAT (C), and POX (D). The error bars are indicated as SD for at least three biological assays.

In sweet potato, wounding stimulates the production of H_2_O_2_ from NADPH oxidase, and generates both local and systemic signals to induce *IPO* expression (Jih *et al.*, 2003). HO, which can product CO, participates in reactive oxygen species scavenging (Balestrasse *et al*., 2008*a*, *b*; Zilli *et al.*, 2009). To investigate the relationship between CO and H_2_O_2_ in wounding responses, H_2_O_2_ content upon wounding was examined in the present of CO (Fig. 4A). The level of H_2_O_2_ was induced after the sweet potato was wounded, whereas it was significantly reduced by pre-supplying the leaves with 5% CO solution.

Previous studies have indicated that CO can activate various antioxidant enzymes (Han *et al.*, 2008; Cui *et al.*, 2012; Xie *et al.*, 2012). The activities of APX, CAT, and POX were then analysed to study how CO inhibits the accumulation of the wound-induced H_2_O_2_. The results showed that CO could influence the activities of APX, CAT, and POX (Fig. 4B–D). Without wounding, CO could significantly increase the CAT activity (time 0 in Fig. 4C) and slightly induce the POX activity (time 0 in Fig. 4D). The activities of APX, CAT, and POX were quickly and intensely induced at 1h after wounding in the present of CO (Fig. 4B–D). Interestingly, APX activity remained higher in the wounded leaves with CO than with water (Fig. 4B). Therefore, CO elevated the activities of APX, CAT, and POX to decompose the H_2_O_2_ induced by wounding and further interfered in *IPO* expression.

### CO suppresses the phosphorylation of ERK induced by wounding

**Fig. 5.**
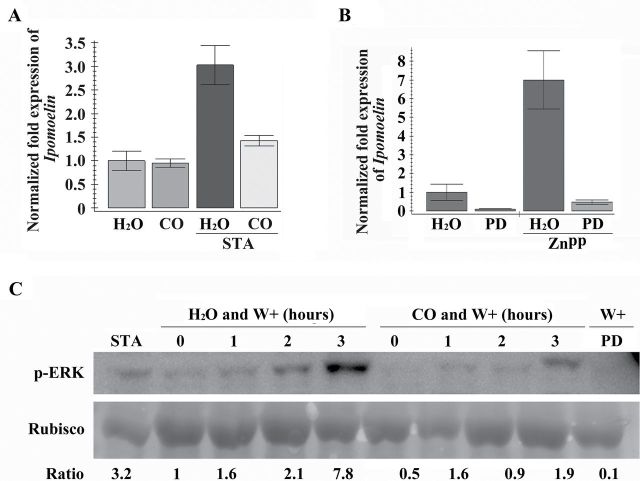
Effects of CO on ERK phosphorylation. (A) Effects of CO on *IPO* expression induced by STA, an ERK1/2 phosphorylation inducer. Leaves with petiole cuts of sweet potato were immersed in water for 12h and then treated with water or 5% CO solution for another 12h. Some of them were treated with 1 μM STA for 2h. Total RNAs from these leaves were analysed by qRT-PCR to detect *IPO* expression. (B) Effects of PD98059, an ERK1/2 phosphorylation inhibitor, on the *IPO* expression induced by ZnPP. Leaves with petiole cuts were immersed in water for 12h and then treated with water or 0.1 μM PD980559 (PD) for another 12h. Some of these leaves were then treated with 10 μM ZnPP for 6h. The *IPO* expression levels of these leaves were analysed by qRT-PCR. *IbActin* expression was used as an internal control. The error bars are indicated as SD for at least three biological assays for both (A) and (B). (C) Effects of CO on the phosphorylation of ERK (p-ERK) upon wounding. Leaves with petiole cuts were immersed in water for 12h and then treated with water, 5% CO solution, or 0.1 μM PD980559 for another 12h. The leaves treated with water and CO were then wounded for 0, 1, 2, and 3h. Leaves treated with PD980559 were further wounded for 6h. Some leaves treated with water were incubated in 1 μM STA for 2h. The total proteins were analysed by western blot assays for the detection of p-ERK. Rubisco from the same amounts of total protein was separated by SDS-PAGE, and stained by Coomassie blue as a loading control.

In animals, the phosphorylation of ERK1/2 can be influenced by CO (Song *et al.*, 2002; Kim *et al.*, 2005; Basuroy *et al.*, 2011; Schallner *et al.*, 2012). MAPK cascades are also involved in the *IPO* induction pathway (Chen *et al.*, 2008). Hence, the potential inducer staurosporine (STA) and inhibitor PD98059 of ERK1/2 phosphorylation were used to examine the effects of CO on ERK phosphorylation. STA induces ERK phosphorylation (Xiao *et al.*, 1999; Cho *et al.*, 2003; Zhao *et al.*, 2013). STA also increases *IPO* expression, whereas PD98059 counteracts STA response for *IPO* induction (Chen *et al.*, 2008). In Fig. 5A, CO blocked the expression of *IPO* induced by STA. In addition, PD98059 inhibited the expression of *IPO* induced by HO repressor ZnPP (Fig. 5B). These results indicated that CO may regulate ERK phosphorylation in sweet potato. Using anti-pERK antibody as a probe in immunoblotting analysis, wounding and STA elevated ERK phosphorylation (Fig. 5C). By contrast, ERK phosphorylation was repressed by CO and PD98059 (Fig. 5C). These results indicated that CO could decrease ERK phosphorylation, and then inhibit *IPO* induction. In wounding responses, therefore, CO contents were decreased to enhance ERK phosphorylation, and then *IPO* expression was induced.

### Identification of ERK of sweet potato

**Fig. 6.**
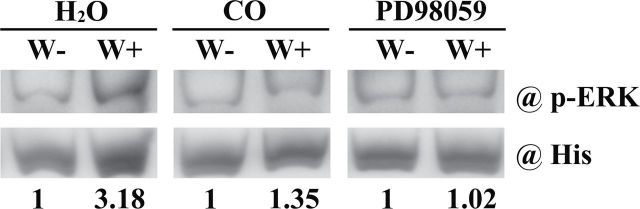
*In vitro* phosphorylation of IbMAPK by anti-p-ERK antibody. Leaves with petiole cuts were immersed in water for 12h, and then treated with water, 5% CO solution, or 0.1 μM PD98059 for another 12h. The leaves were then left unwounded (W-) or wounded (W+) by tweezers. The total proteins extracted from these leaves were incubated with recombinant IbMAPK–His at 30 °C for 1h and purified by His resin. The bound proteins were eluted from the resin and detected by anti-p-ERK and anti-His antibody. Immunoblots using anti-His antibody were used as controls.

Anti-pERK antibody mainly detects a short amino acid sequence containing phosphorylated Tyr 204 of ERK 1 of human origin (http://www.scbt.com/datasheet-7383-p-erk-e-4-antibody.html), and the activation of ERK1/2 is inhibited in the presence of PD98059 (Alessi *et al.*, 1995; Gudesblat *et al.*, 2007). MPK3 and MPK6 of *Arabidopsis* can be detected by anti-pERK antibody in immunoblotting analyses (Singh *et al.*, 2012; Gonzalez Besteiro and Ulm, 2013). Hence, ERK of sweet potato was searched using bioinformatics. An IbMAPK (GenBank accession no. AAD37790) was obtained by BLAST using the conserved domain of MPK3 and MPK6 as the search criterion, and was homologous to the MPK3 proteins from *Arabidopsis*, tomato, and tobacco (Supplementary Fig. S3A at *JXB* online). A phylogenetic analysis of these proteins was performed (Supplementary Fig. S3B). The recombinant protein of IbMAPK was further purified using a bacterial expression system. Phospho-IbMAPK could also be detected by anti-pERK antibody as a probe (Fig. 6). The total proteins from wounding leaves could elevate the phosphorylation of the recombinant IbMAPK protein, whereas those from CO- and PD98059-pre-treated wounding leaves could not (Fig. 6). Thus, this IbMAPK was the ERK involved in the wounding responses of sweet potato.

### Interaction between IbMEK1 and IbMAPK *in vivo* and *in vitro*

**Fig. 7.**
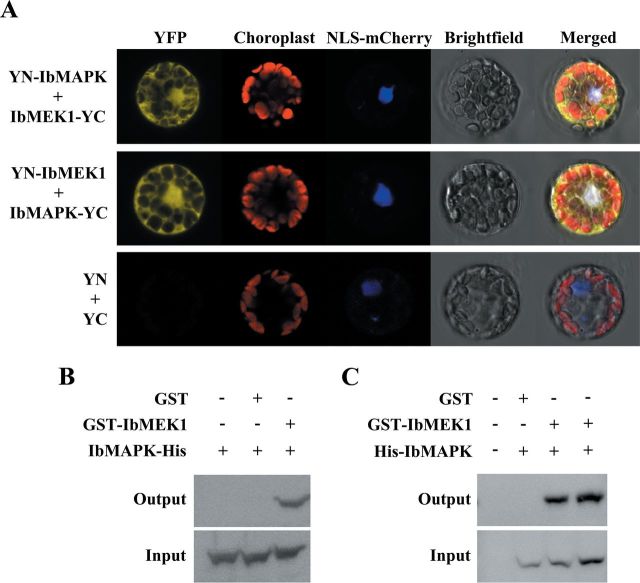
IbMEK1 interacts with IbMAPK both *in vivo* and *in vitro*. (A) BiFc assays in *Arabidopsis* protoplasts for interaction between IbMEK1 and IbMAPK. Protoplasts were co-transformed with plasmids encoding IbMEK1 and IbMAPK fused with the YC and YN of YFP. These protoplasts were then visualized using a confocal microscope. Column 1 shows signals from YFP, column 2 shows chlorophyll autofluorescence, column 3 shows signals from NLS–mCherry as a nuclear maker, column 4 shows bright-field images, and column 5 shows merged images of columns 1–4. (B) GST pull-down assays for interaction between IbMEK1 and IbMAPK. GST–IbMEK1 or GST was incubated with IbMAPK–His and GST resin, and the bound proteins were then eluted from resin. These eluted proteins were detected with an anti-His antibody. (C) Co-immunoprecipitation assays in *N. benthamiana* leaves for interaction between IbMEK1 and IbMAPK. Tobacco leaves were infiltrated with agrobacteria carrying vectors containing *35S*:*GST* (GST), *35S*:*GST-IbMEK1* (GST–IbMEK1), or *35S*:*His-IbMAPK* (His–IbMAPK). After 4 d, total proteins extracted from these infiltrated leaves were incubated with GST resin, and the bound proteins were then eluted from the resin. These eluted proteins were detected with an anti-His antibody.

PD98059 can inactivate MEK1/2, and further inhibits p-ERK activation. Hence, the IbMEK1 of sweet potato was also identified by bioinformatics using the conserved domain of MEK1/2 from other plants as the search criterion, and its full-length sequence was obtained by RACE. IbMEK1 was homologous to the MEK1/2 proteins from *Arabidopsis*, tomato, and rice (Supplementary Fig. S4A at *JXB* online), and a phylogenetic analysis of these proteins was performed (Supplementary Fig. S4B). BiFC (Fig. 7A), GST pull-down assays (Fig. 7B), and co-immunoprecipitation (Fig. 7C) all showed that IbMEK1 could interact with IbMAPK, indicating the interaction between IbMEK1 and IbMAPK would occur both *in vivo* and *in vitro*. In wounding responses, taken together, IbMEK1 might activate the phosphorylation of IbMAPK.

## Discussion

HO converts haem to biliverdin, CO, and free iron (Muramoto *et al.*, 2002; Terry *et al.*, 2002; Shekhawat and Verma, 2010). Biliverdin and CO participate in reactive oxygen species scavenging though activating antioxidant enzymes (Xie *et al.*, 2012). The addition of biliverdin or CO partially rescues the UV-C hypersensitivity responses in the *hy1* mutant (Xie *et al.*, 2012). CO also partially rescues the phenotypes of *SE5*-RNAi plants in the herbicide paraquat-induced oxidative stress (Xu *et al.*, 2012). The addition, HO products, especially CO, can mimic the responses of root development after treatment of Hm, salt, or polyethylene glycol (Cao *et al.*, 2011). CO also plays an important signal in seed germination (Dekker and Hargrove, 2002), iron homeostasis (Kong *et al.*, 2010), and various stress defences (Yannarelli *et al.*, 2006; Xie *et al.*, 2008; Shen *et al.*, 2011; Cui *et al.*, 2012). In this study, CO contents were decreased in sweet potato after wounding (Fig. 1A), indicating that CO might act as a negative physiological messenger to regulate downstream genes.

HO has been identified from different plants and proven to play important roles in plant developmental processes and stress responses. IbHO1 shared significant similarity with other known HO1 proteins in plants (Supplementary Fig. S1a). In *Arabidopsis*, HO includes the HO1 and HO2 subfamilies based on sequence similarity. However, HO2 lacks the canonical HO activate site, a positionally conserved histidine, and is thus considered a fake HO (Snyder and Baranano, 2001; Gisk *et al.*, 2010). IbHO1 contained the conserved haem interaction residues and a His haem ligand (Supplementary Fig. S1). Based on the sequence similarity, IbHO1 is a member of the HO1 protein, which has been considered a stress response protein in plants. GmHO1 has been found to be significantly induced by UV-B irradiation and salinity stresses (Xie *et al.*, 2012). AtHO1 was induced by iron deficiency in *Arabidopsis* (Kong *et al.*, 2010). After wounding, IbHO1 was significantly repressed at the early intervals of 0.5 and 1h (Fig. 1B), and the main reduction in CO was detected at 3h after wounding (Fig. 1A). Therefore, IbHO1 and its product CO may participate in the wounding response.

IPO has been characterized as a defence-related protein in sweet potato. In the wounding response, IPO can be induced to inhibit silkworm growth and survival rates (Chen *et al.*, 2005). Hm functioned in a concentration-dependent manner to inhibit the *IPO* expression induced by wounding (Fig. 2A). In wheat and rapeseed, CO contents were elevated through the HO activated by Hm (Cao *et al.*, 2007; Xuan *et al.*, 2007). The treatment of CO or Hm showed similar functions in various phenotypes including root development and antioxidant activation (Cao *et al.*, 2011; Xu *et al.*, 2012). In addition to the effect of Hm, *IPO* induction was also repressed in the presence of CO (Fig. 3A). ZnPP, an HO inhibitor, was used in cucumber root, broad bean, and wheat aleuronic layers to inhibit HO activity and further decrease the production of CO (Song *et al.*, 2008; Xuan *et al.*, 2008*b*; Wu *et al.*, 2011). ZnPP was also used here to inhibit HO activity to decrease CO production. In addition, the more ZnPP was added, the more *IPO* expression was observed (Fig. 2B). In conclusion, the *IPO* expression was inhibited by CO, which was generated from HO. After wounding, HO activity was decreased, the amount of CO was reduced, and *IPO* was then activated.

In plants, H_2_O_2_ is involved in redox signalling to regulate local and distal wound signals (Orozco-Cardenas and Ryan, 1999; Orozco-Cardenas *et al.*, 2001), and it also activates wound-induced genes to protect plants from pathogen and insect attacks (Moloi and van der Westhuizen, 2006; Choi *et al.*, 2007). In sweet potato, wounding stimulates the production of H_2_O_2_ from NADPH oxidase and generates both local and systemic signals to induce *IPO* expression (Jih *et al.*, 2003). MJ also stimulates the production of H_2_O_2_ (Orozco-Cardenas *et al.*, 2001), and further induces *IPO* expression (Jih *et al.*, 2003). The addition of CO inhibited MJ- or H_2_O_2_-induced *IPO* expression (Fig. 3B), and significantly decreased H_2_O_2_ contents (Fig. 4A). Hence, CO might regulate H_2_O_2_ contents to affect *IPO* induction. The excess production of H_2_O_2_ acts as an oxidative stress to mediate chlorophyll decay, lipid peroxidation, ion leakage, and DNA and protein modification (Halliwell and Gutteridge, 1984; Mittler, 2002; Yang *et al.*, 2006). Thus, the balance of H_2_O_2_ production in cells is very complex and important to ensure that organisms and cellular components work well (Foyer and Noctor, 2005). Furthermore, in the wheat aleuronic layer, gibberellin stimulates H_2_O_2_ production and further gives rise to programmed cell death. The application of exogenous CO significantly decreases the production of H_2_O_2_ and prevents gibberellin-induced programmed cell death (Wu *et al.*, 2011). In cadmium-induced oxidative stress, CO induces various antioxidant activities, including SOD, CAT, APX, and POX, to scavenge reactive oxygen species (Cui *et al.*, 2012). In soybean, HO1 activates SOD, CAT, and APX to protect plant cells against UV-C irradiation (Xie *et al.*, 2012). The activities of CAT, APX, and POX were also elevated by CO in wounding responses (Fig. 4B). Therefore, CO could activate APX, CAT, and POX to scavenge the H_2_O_2_ induced by wounding, and further interfered in *IPO* expression.

MAPK cascades are the major pathways to drive extracellular stimuli to multiple intercellular responses in mammals, yeast, and plants. The kinases in MAPK cascades of plants also share significant similarity with the kinase families found in animals (Colcombet and Hirt, 2008). In *Arabidopsis*, 20 MAPKs, 10 MAPKKs, and 80 MAPKKKs have been found based on the genomic sequence databases (Colcombet and Hirt, 2008). Among the MAPKs, AtMPK3 and AtMPK6 can be detected by anti-pERK antibody in immunoblotting analyses (Lee and Ellis, 2007; Singh *et al.*, 2012). ERK1/2 activation in mammalian cells has been reported to trigger cell death (Stanciu *et al.*, 2000). MPK3 and MPK6 have also been shown to be associated with the stress-induced cell death in plants (Lee and Ellis, 2007; Colcombet and Hirt, 2008), and regulate the levels of jasmonic acid and SA in various stresses (Yang *et al.*, 2001; Menke *et al.*, 2004; Seo *et al.*, 2007). MAPK cascades play important roles in the induction of *IPO* expression (Chen *et al.*, 2008). In animals, CO affects the phosphorylation of ERK1/2 (Song *et al.*, 2002; Kim *et al.*, 2005; Basuroy *et al.*, 2011; Schallner *et al.*, 2012). The phosphorylation of IbMAPK, the orthologue of AtMAPK3, was also affected by CO in wounding responses (Figs 5C and 6). PD98059 can prevent MEK1 activity and then block its downstream activity (Burnett *et al.*, 2000; Xing *et al.*, 2008; Li *et al.*, 2012), and blocked IbMAPK phosphorylation in the wounding response (Fig. 5). These results indicated that MEK1 may activate IbMAPK. BiFC, co-immunoprecipitation, and GST pull-down assays also identified the interaction between IbMEK1 and IbMAPK (Fig. 7). Taken together, CO might prevent IbMAPK from IbMEK1 activation. Wounding could decrease CO content to activate the phosphorylation of IbMAPK through IbMEK1.

**Fig. 8.**
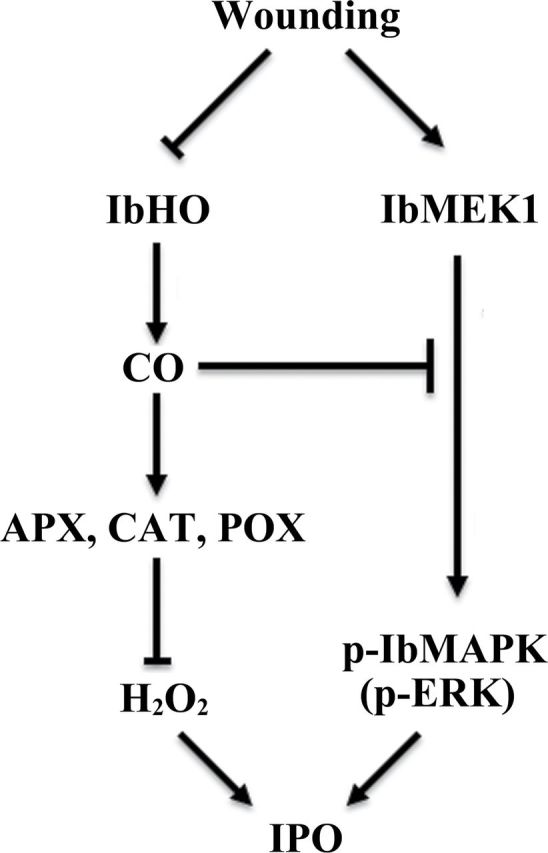
Schematic representation of the role of CO in the signal transduction pathway inducing *IPO* expression. The signal transducers, including H_2_O_2_ and MAPK cascades, induced by wounding for stimulating *IPO* expression have been reported previously (Chen *et al.*, 2003, 2008). In sweet potato, CO could increase the activities of antioxidants APX, CAT, and POX and decrease the accumulation of H_2_O_2_ and the phosphorylation of ERK. Upon wounding, the expression of *IbHO* was repressed, and further decreased the contents of CO. Subsequently, the content of H_2_O_2_ and the phosphorylation of ERK were induced, and the wound-inducible gene *IPO* was then expressed.

Conclusively, CO produced by HO may act as a negative regulator to inhibit *IPO* expression in sweet potato (Fig. 8). CO could induce the activities of CAT, APX, and POX to reduce the H_2_O_2_ production (Fig. 4A–D). The phosphorylation of IbMAPK was also inhibited by CO (Fig. 5C). When leaves were wounded, the HO and CO contents were reduced (Fig. 1A, B). H_2_O_2_ is then produced, IbMAPK phosphorylation is elevated, and *IPO* expression is finally induced (Fig. 8).

## Supplementary data

Supplementary data is availabel at *JXB* online.

Supplementary Table S1. Primers for this study.

Supplementary Fig. S1. Protein sequence comparisons and the phylogenetic analyses of haem oxygenase (HO).

Supplementary Fig. S2. CO contents in the leaves of sweet potato treated with Hm or ZnPP.

Supplementary Fig. S3. Protein sequence comparisons and the phylogenetic analyses of MAPK.

Supplementary Fig. S4. Protein sequence comparisons and the phylogenetic analyses of MEK.

Supplementary Data

## Abbreviations:

APX: ascorbate peroxidase
BiFC: bimolecular fluorescence complementation
CAT: catalase
CO: carbon monoxide
ERK: extracellular signal-regulated kinase
GST: glutathione *S*-transferase
Hb: haemoglobin
Hm: hemin
HO: haem oxygenase
IPO: ipomoelin
MAPK: mitogen-activated protein kinase
MJ: methyl jasmonate
NO: nitric oxide
POX: peroxidase
qRT-PCR: quantitative reverse transcription-PCR
RACE: rapid amplification of cDNA ends
SA: salicylic acid
SD: standard deviation
SOD: superoxide dismutase
STA: staurosporine
YFP: yellow fluorescent protein
ZnPP: zinc protoporphyrin IX.
