# Supplementary material for: Carbon monoxide regulates the expression of the wound-inducible gene ipomoelin through antioxidation and MAPK phosphorylation in sweet potato
Source: J Exp Bot. 2014 Jul 25;65(18):5279–90. doi: 10.1093/jxb/eru291 (PMC4157712; doi:10.1093/jxb/eru291)
Supplement: Supplementary Data [file supp_65_18_5279__index.html]

Carbon monoxide regulates the expression of the wound-inducible gene ipomoelin through antioxidation and MAPK phosphorylation in sweet potato — Carbon monoxide regulates the expression of the wound-inducible gene ipomoelin through antioxidation and MAPK phosphorylation in sweet potato — Supplementary Data 

# Carbon monoxide regulates the expression of the wound-inducible gene *ipomoelin* through antioxidation and MAPK phosphorylation in sweet potato

## Supplementary Data

Data files

**Files in this Data Supplement:**

- Supplementary Data - Supplementary Data
- Supplementary Data - Supplementary Data
- Supplementary Data - Supplementary Data
