# Supplementary material for: Carbon monoxide regulates the expression of the wound-inducible gene ipomoelin through antioxidation and MAPK phosphorylation in sweet potato
Source: J Exp Bot. 2014 Jul 25;65(18):5279–90. doi: 10.1093/jxb/eru291 (PMC4157712; doi:10.1093/jxb/eru291)
Supplement: Supplementary Data [file supp_eru291_jexbot125096_file001.pdf]

Supplementary Table

Table S1. Primers for this study

$T_{25}VN$	TTTTTTTTTTTTTTTTTTTTTTTTTVN
IPO F	GCTTAATCCTCCATTGGAACACCTCTAT
IPO R	ACATCATATCACACTAGACGAGACGACAA
IbHO1 F	GGAGAAGGAGACTGAAGGCAAGC
IbHO1 R	GCGACCTTTCTAATCCCGTGTTCC
IbActin F	GACTACCATGTTCCCCGGTA
IbActinR	TTGTATGCCACGAGCATCTT
IbMAPK F	CGGATTGCTGACATCACCATG
IbMAPK R	TTATGCATATCCTGTATTCATTGCCA
IbMEK1 F	GCTGTGGTGGGCAATATGGGAAA
IbMEK1 R	TCA AAATGCTGCACGTC
XbaI-IbMAPK F	TCTAGACGGATTGCTGACATCACCATG
BamHI-IbMAPK R	GGATCCTGCATATCCTGGATTCATTGC
BamHI-IbMAPK F	GGATCCATGGTGGGCGGCGGC
SacI-IbMAPK R	GAGCTCTTATGCATATCCTGTATTCATTG
HindIII-IbMAPK R	AAGCTTATGCATATCCTGTATTCATTG
BamHI-IbMEK1 F	GGATCCATGAAGAAAGGAGCTTTAG
HindIII-IbMEK1 R	AAGCTTAAATGCTGCACGTCGAGGTC
XbaI-IbMEK1 F	TACAGAATGAAGAAAGGAGCTTTAGCCC
BamHI-IbMEK1 R	GGATCCAAATGCTGCACGTCGAGG
SacI-IbMEK1 R	GAGCTCTCAAAATGCTGCACGTCGAGG
EcoRI-IbMEK1 R	GAATTCTCA AAATGCTGCACGTCGAGG
BamHI-GST F	GGATCCATGTCCCCTATACTAGGTTATTGG
SacI-GST R	GAGCTCTTACAGGGGCCCCTGGACAG

Supplementary Figures

A

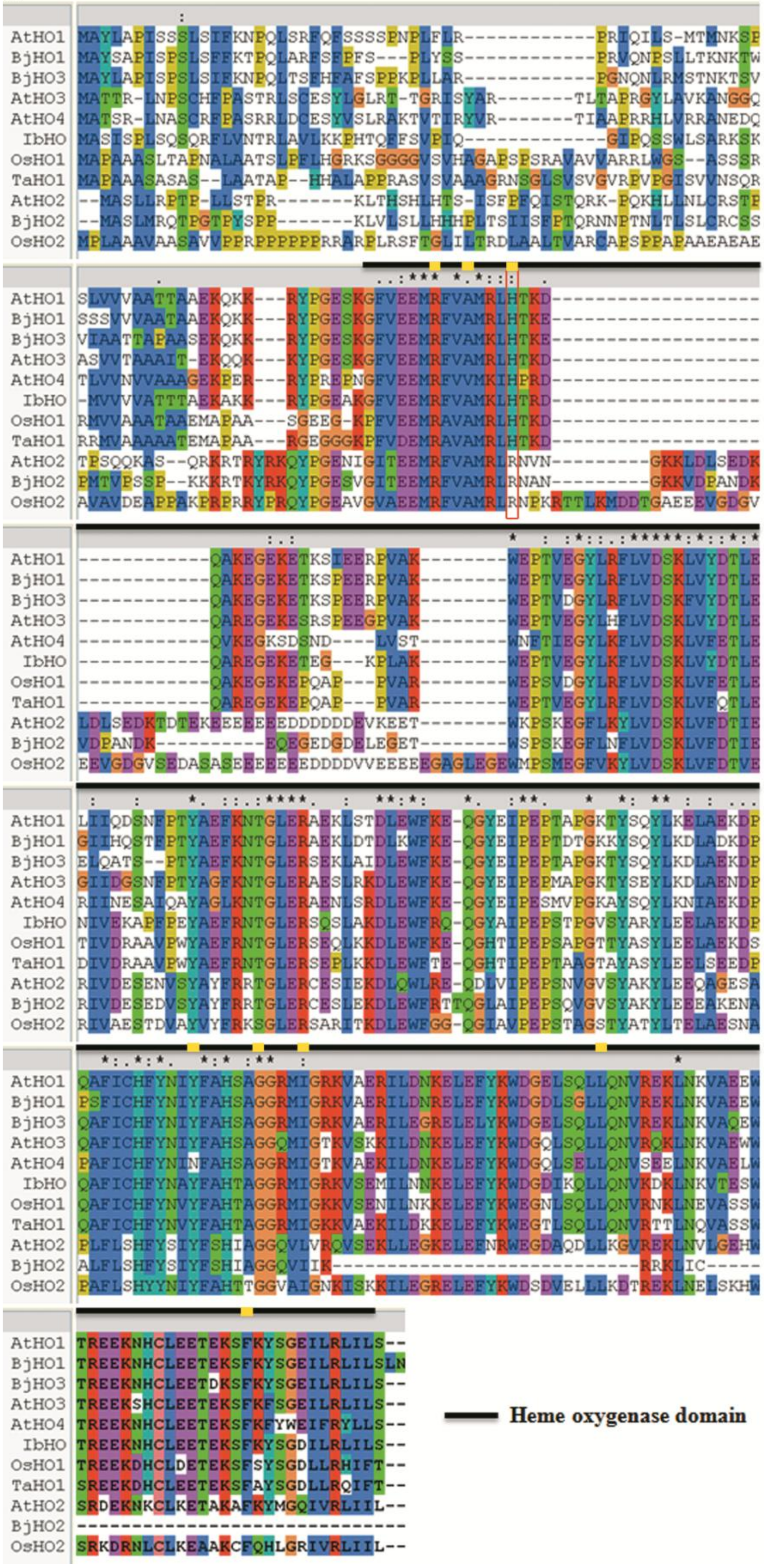

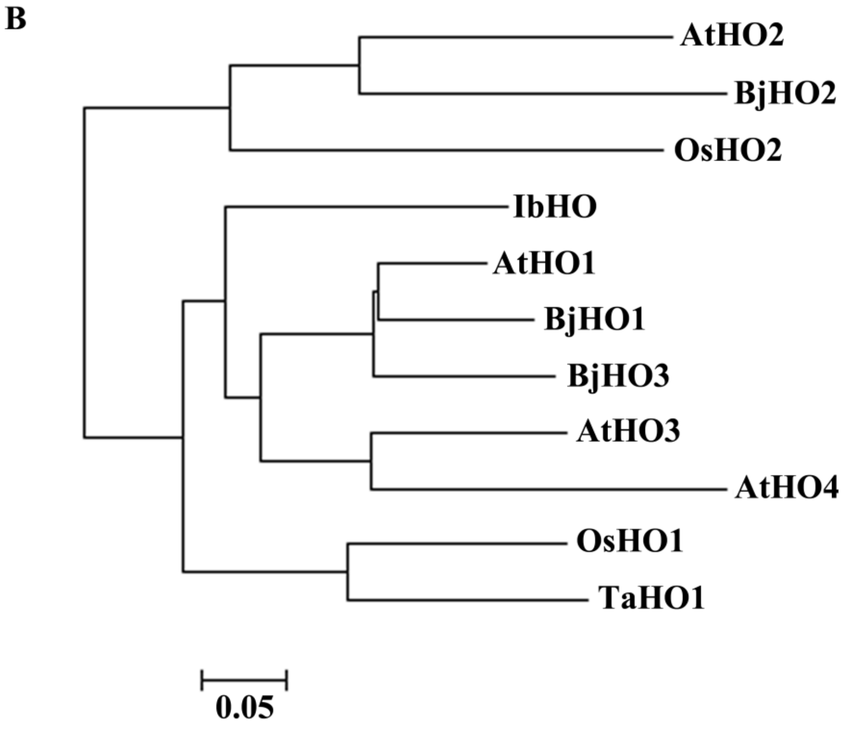

Figure S1. Protein sequence comparisons and the phylogenetic analyses of heme oxygenases (HO)

(A) Protein sequence comparisons were produced by ClustalX2. IbHO was compared with those encoding similar proteins in Arabidopsis, rice, *Triticum aestivum*, and *Brassica juncea*, which include AtHO1 (NP_180235), AtHO2 (NP_001189610), AtHO3 (NP_177130), AtHO4 (NP_176126), OsHO1 (NP_001058011), OsHO2 (NP_001050290), TaHO1 (AEI69674), BjHO1 (AET97566), BjHO2 (AET97567), and BjHO3 (AET97568). '*' indicates identical amino acid residues in all sequences, ':' indicates highly conserved amino acid residues, and '.' indicates weakly conserved amino acid residues. The lines above letters indicate heme oxygenase domain. The yellow lines indicate heme binding pockets. The red box indicates the histidine heme ligand. The interpretation of the color backgrounds of amino acid residues is listed in http://ekhidna.biocenter.helsinki.fi/pfam2/clustal_colours. (B) The phylogenetic trees were then constructed using neighbor-joining method with MEGA 5.1 program.

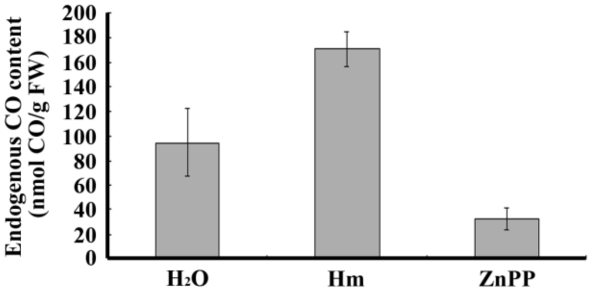

Figure S2. CO contents in the leaves of sweet potato treated with Hm or ZnPP

Sample treatments are the same as described in the legend of Figure 2. Leaves with petiole cuts of sweet potato were immersed in water for 12 hours, and then treated with 10 μM Hm for another 18 hours. In addition, leaves with petiole cuts were immersed in water for 24 hours, and treated with 10 μM ZnPP for another 6 hours. The endogenous CO levels of these leaves were detected by hemoglobin binding assays. The error bars are indicated as the standard deviation for at least three biological assays.

A

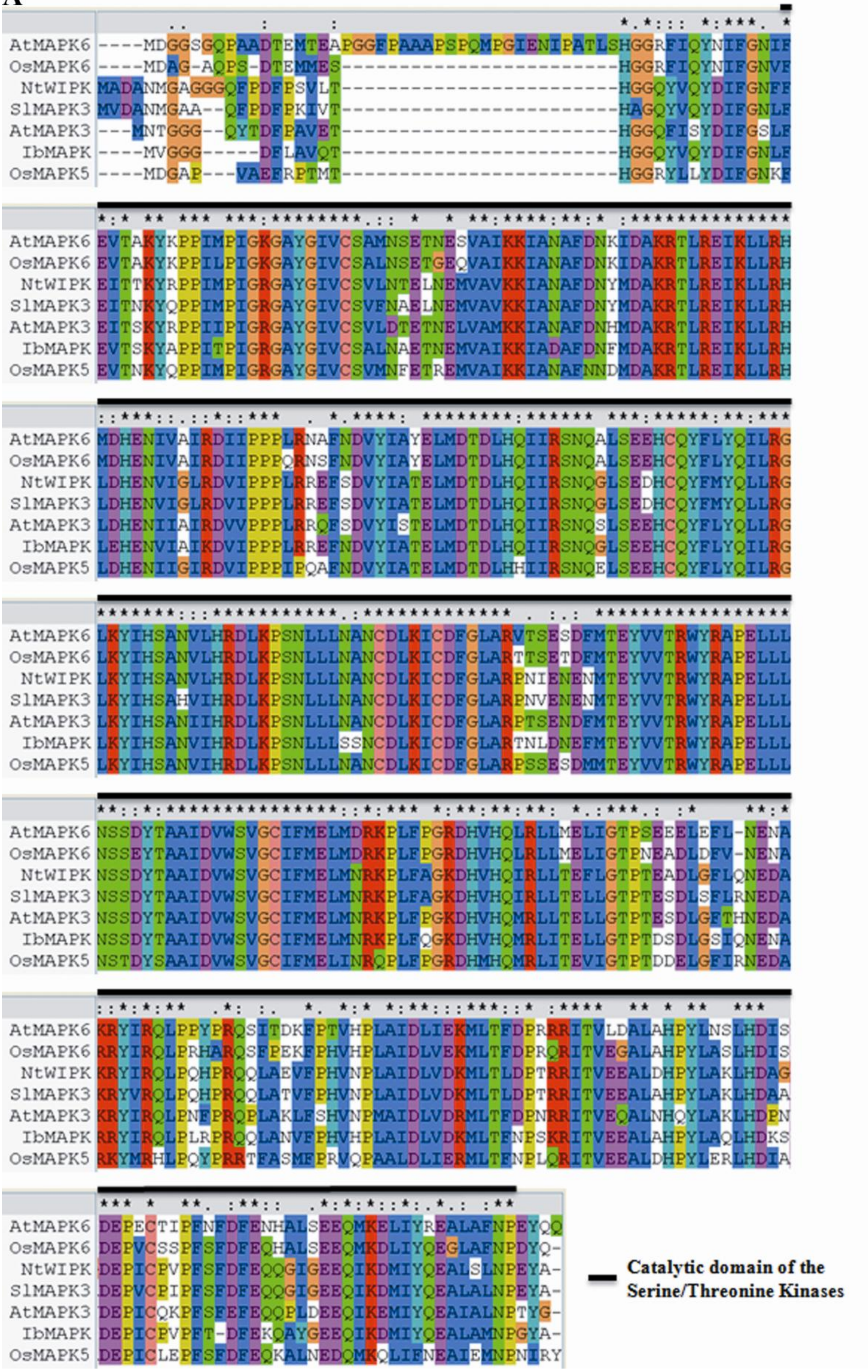

B

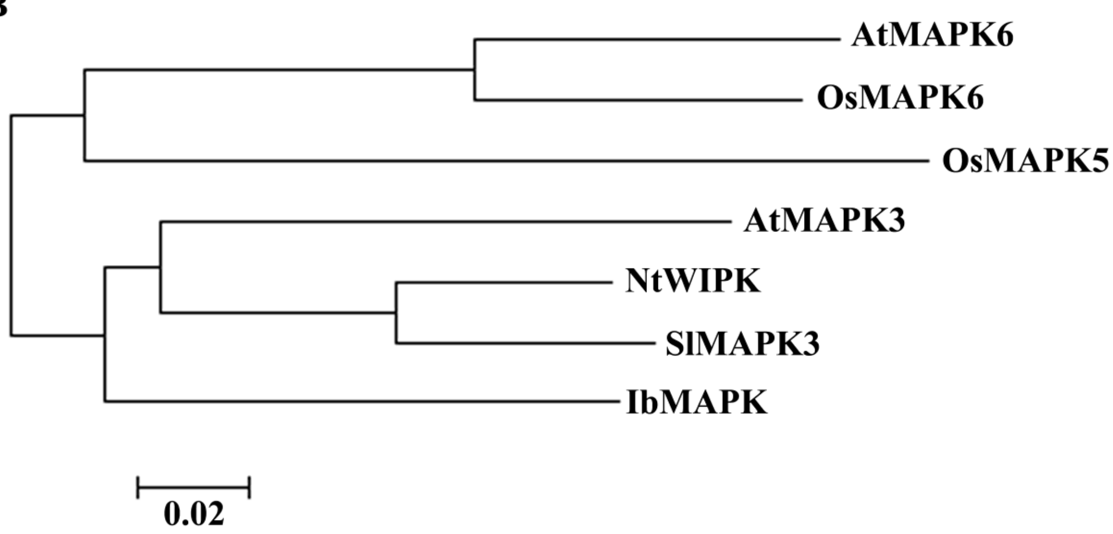

Figure S3. Protein sequence comparisons and the phylogenetic analyses of MAPK (A) Protein sequence comparisons were produced by ClustalX2. IbMAPK (AAD37790) was compared with those encoding similar proteins in Arabidopsis, *Nicotiana tabacum*, *Solanum lycopersicum*, and rice, which included AtMAPK3 (NP_190150), AtMAPK6 (NP_181907), NtWIPK (BAB79636), SlMAPK3 (NP_001234360), OsMAPK5 (NP_001049770), and OsMAPK6 (ACD76439). '*' indicates identical amino acid residues in all sequences, ':' indicates highly conserved amino acid residues, and '.' indicates weakly conserved amino acid residues. The black lines above letters indicate the catalytic domain of the serine/threonine kinases. The interpretation of the color backgrounds of amino acid residues is listed in http://ekhidna.biocenter.helsinki.fi/pfam2/clustal_colours. (B) The phylogenetic trees were then constructed using neighbor-joining method with MEGA 5.1 program.

A

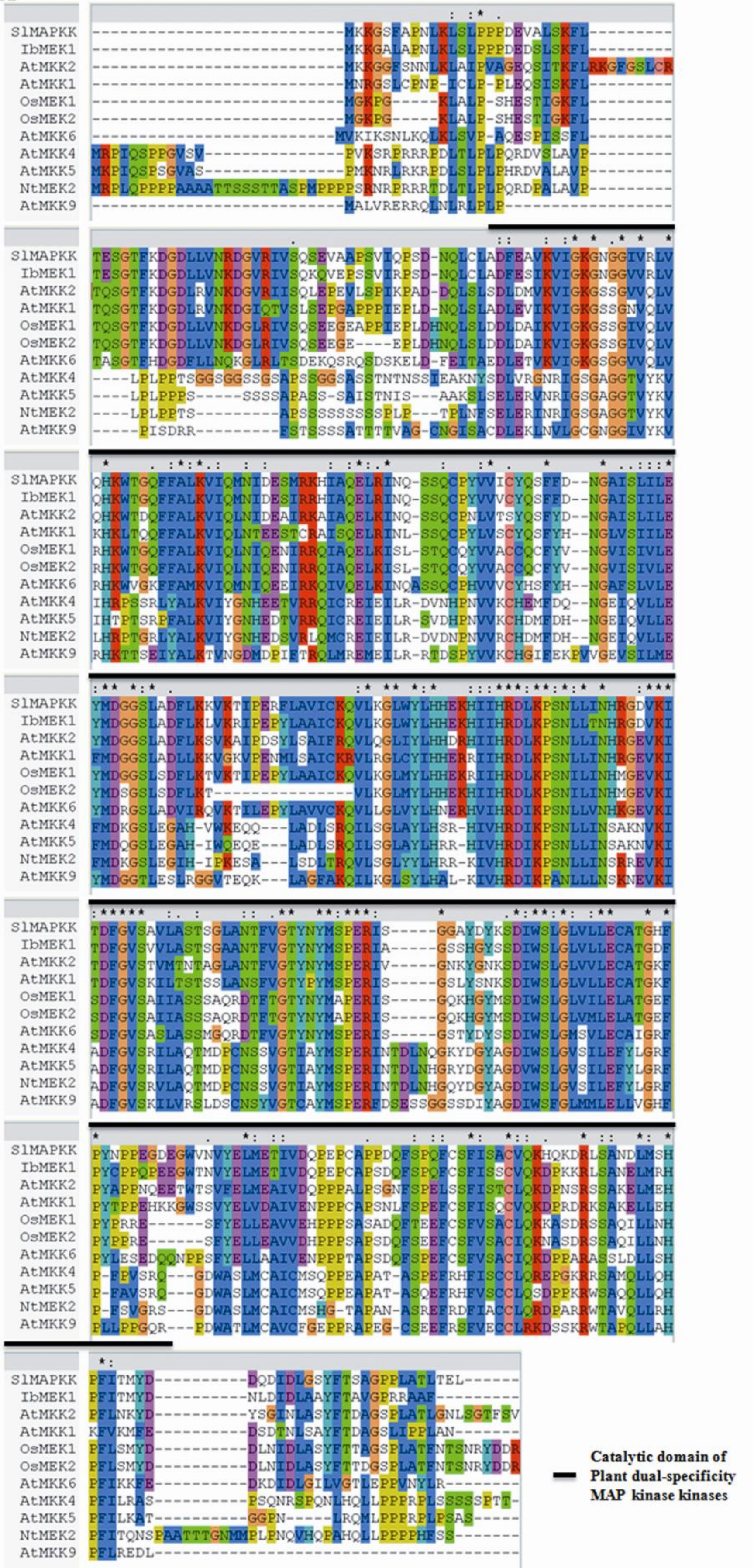
Catalytic domain of
Plant dual-specificity
MAP kinase kinases

B

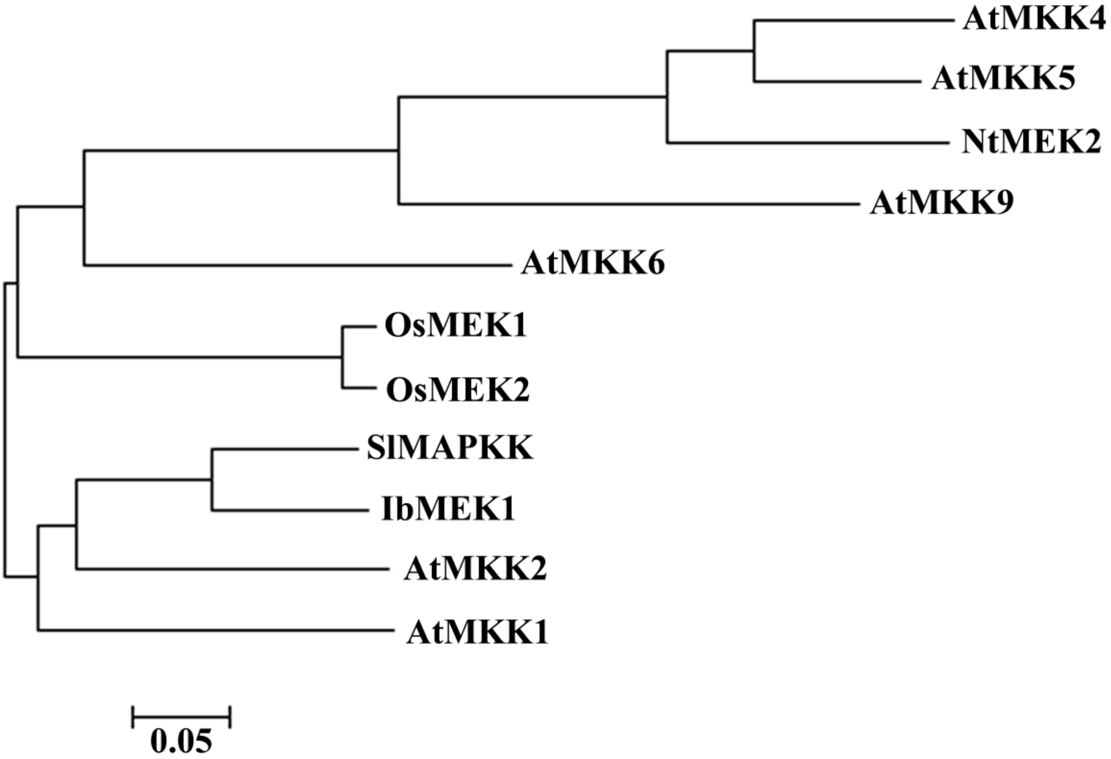

Figure S4. Protein sequence comparisons and the phylogenetic analyses of MEK (A) Protein sequence comparisons were produced by ClustalX2. IbMEK1 was compared with those encoding similar proteins in Arabidopsis, *Nicotiana tabacum*, *Solanum lycopersicum*, and rice, which included AtMKK1 (NP_194337), AtMKK2 (BAH19880), AtMKK4 (ABF55664), AtMKK5 (ABF55665), AtMKK6 (ABF55666), AtMKK9 (NP_177492), NtMEK2 (AAG53979), SlMAPKK (AAU04433), OsMEK1 (ABP88102), and OsMEK2 (CAD45180). '*' indicates identical amino acid residues in all sequences, ':' indicates highly conserved amino acid residues, and '.' indicates weakly conserved amino acid residues. The black lines above letters indicate the catalytic domain of plant dual-specificity MAP kinase kinases. The interpretation of the color backgrounds of amino acid residues is listed in http://ekhidna.biocenter.helsinki.fi/pfam2/clustal_colours. (B) The phylogenetic trees were then constructed using neighbor-joining method with MEGA 5.1 program.
