# Supplementary figures and images for: Carbon monoxide regulates the expression of the wound-inducible gene ipomoelin through antioxidation and MAPK phosphorylation in sweet potato
Source: J Exp Bot. 2014 Jul 25;65(18):5279–90. doi: 10.1093/jxb/eru291 (PMC4157712; doi:10.1093/jxb/eru291)

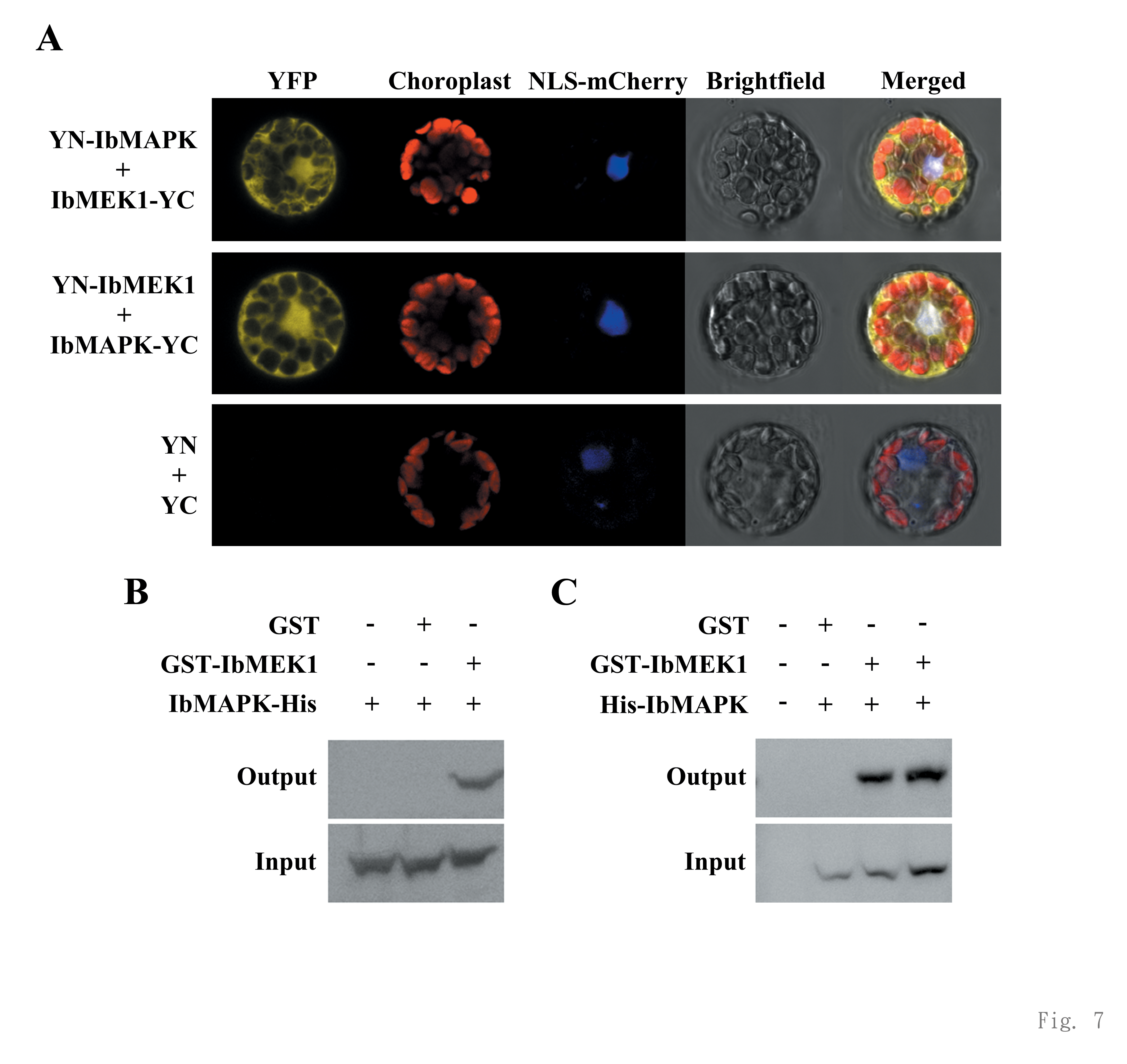

Supplement: Supplementary Data [file supp_eru291_jexbot125096_file002.tif]

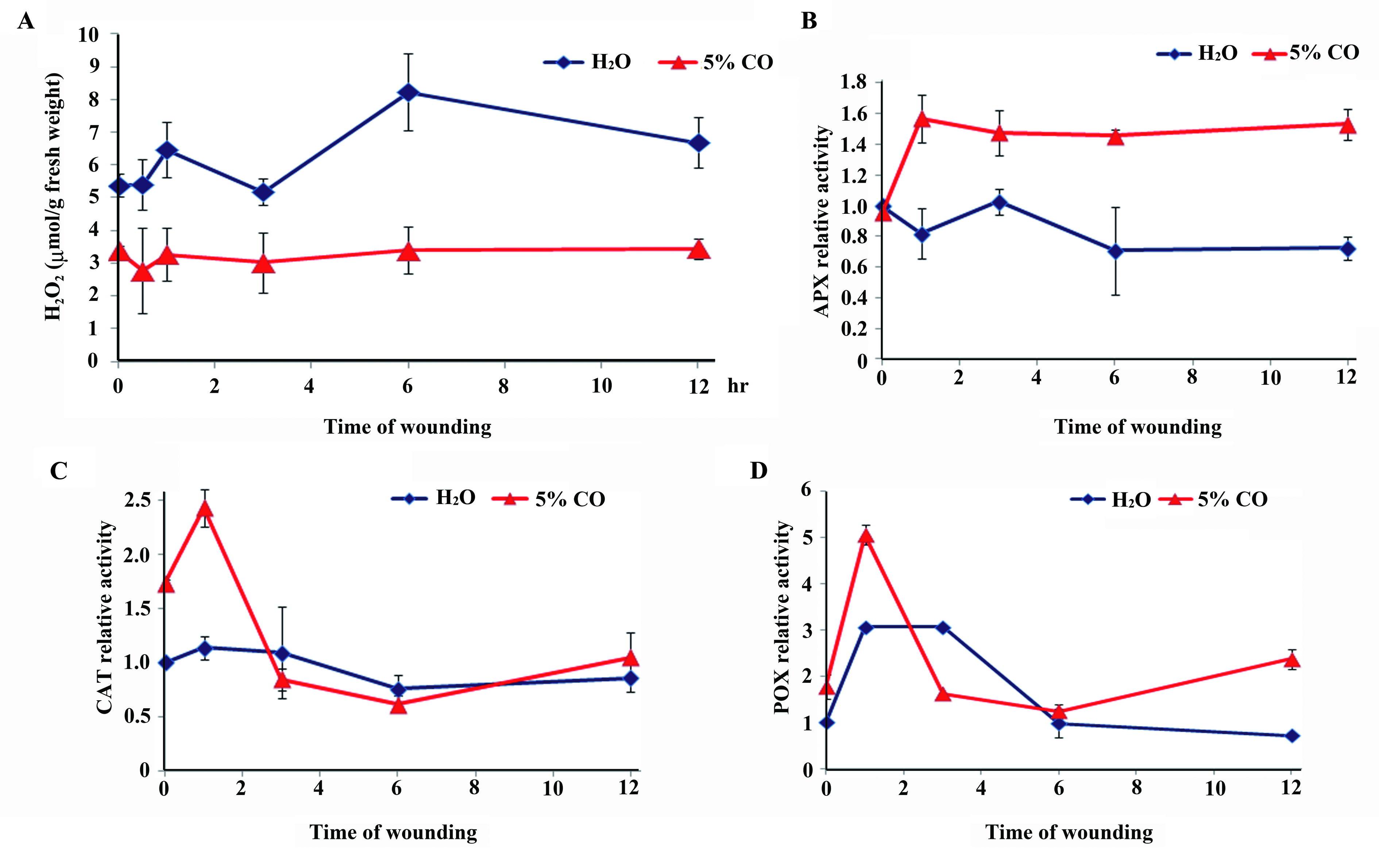

Supplement: Supplementary Data [file supp_eru291_jexbot125096_file003.tif]
